# Computational Reconstruction of Integral Imaging for Accurate Depth Estimation Using Non-Uniform Pitches

**DOI:** 10.3390/s26216827

**Published:** 2026-10-29

**Authors:** Myungjin Cho, Byungwoo Cho, Jungsik Koo

**Affiliations:** 1 School of ICT, Robotics, and Mechanical Engineering, IITC, Hankyong National University, 327 Chungang-ro, Anseong 17579, Gyeonggi-do, Republic of Korea; 2 Gumi Electronics & Information Technology Research Institute, 8-26, Gumi-daero 32-gil, Gumi-si 39253, Gyeongsangbuk-do, Republic of Korea; bwcho@geri.re.kr (B.C.); jskoo@geri.re.kr (J.K.)

**Keywords:** depth estimation, integral imaging, non-uniform pitches, shifting pixels, approximate squared-multiple rule, volumetric computational reconstruction

## Abstract

Integral imaging is a promising technology for passive three-dimensional (3D) scene acquisition and display, providing full parallax and continuous viewpoints without the need for specialized viewing glasses. However, in volumetric computational reconstruction (VCR), depth estimation accuracy is basically constrained by shifting pixel quantization errors arising from the discrete pixelation of elemental images. Although non-uniform VCR (NVCR) can reduce these errors through localized rounding, shifting pixel quantization errors may not be reduced to exactly zero. In this paper, we propose a computational reconstruction method for integral imaging that completely eliminates shifting pixel quantization errors while preserving uniform depth resolution across multiple target depths. The proposed method uses a least common multiple (LCM) search based on the respective optimum pitch bases of multiple target planes to calculate a unified optimum pitch. Simultaneously, the physical layout of the camera array is structured non-uniformly using a Latin-square distribution of subarrays governed by the approximate squared-multiple rule. This layout maintains a constant depth resolution limit across the entire distance range and avoids the quadratic degradation of depth resolution observed at farther distances. Through mathematical analysis and computational simulations, we demonstrate that the proposed non-uniform pitch method achieves exact depth recovery with a mean squared error of zero (MSE = 0) at all target depths and produces sharp, high-intensity peak sidelobe ratio (PSR) responses. This approach holds significant potential for advanced sensing applications, such as autonomous vehicles, digital twins, and industrial robotics.

## 1. Introduction

Accurate acquisition of three-dimensional (3D) scene and objects is a critical component of a wide range of modern industries, such as unmanned autonomous vehicles, medical imaging, defense, and advanced manufacturing. To reliably extract and utilize this spatial information, high-precision 3D imaging technologies are required. Among these technologies, integral imaging [1,2,3,4,5,6,7,8,9] has emerged as a promising passive 3D acquisition technique that utilizes a lens or camera array under passive illumination, eliminating the need for coherent light sources. During the acquisition stage, integral imaging records multiple two-dimensional (2D) perspective views of a 3D scene, known as elemental images. During reconstruction, these elemental images can be displayed through a homogeneous lens array to optically reconstruct the 3D scene, providing full parallax and continuous viewpoints without specialized viewing glasses.

Alternatively, 3D depth information can be digitally reconstructed using computational algorithms such as volumetric computational reconstruction (VCR) [10,11,12,13,14]. The VCR estimates a 3D depth map of an object or scene by shifting and summing the elemental images acquired during the acquisition stage. Because VCR operates within a discrete digital domain, shifts must be quantized to integer values to align with the pixel grid of the processing system. This quantization inevitably introduces depth errors [14]. Traditional fixed VCR algorithms [11,12] shift each elemental image with a uniform, integer-quantized base shift. As a result, a cumulative quantization error develops between the actual continuous shifting pixel sequence and the forced integer multiples as the elemental image index increases, leading to inaccurate depth estimation and a degraded resolution profile.

To reduce this cumulative quantization error, non-uniform VCR (NVCR) was proposed [14]. Rather than using a single integer multiplier for all elemental images, NVCR calculates an individually rounded pixel shift for each elemental image. By applying localized rounding, the NVCR reduces the average quantization error and enhances the sharpness of the estimated depth compared to the fixed VCR. Nevertheless, NVCR may not reduce the quantization error to exactly zero because individual shifts remain constrained to discrete integer values. To achieve a zero quantization error, the physical system parameters must be matched to the reconstruction depth plane so that the actual real-valued shifts resolve exactly to integers. This requirement establishes the concept of an optimum pitch basis [15], of which any general optimum pitch is an integer multiple.

In conventional integral imaging, the camera array is constructed with a uniform pitch, as in synthetic aperture integral imaging (SAII) [16], to obtain high-resolution elemental images. However, because the optimum pitch basis is a direct mathematical function of specific object distances which have an integer shifting pixel value, a uniform array can satisfy the zero quantization error condition for these specific object distances. If a 3D scene contains multiple objects at different depths, a uniform pitch array may not optimize all planes simultaneously; significant quantization errors and focal blur therefore recur at off-target distances. Resolving this limitation requires a unified optimum pitch method that accommodates multiple target depths throughout the reconstructed volume.

Moreover, even when an optimum pitch is determined to satisfy the integer-shift condition at multiple depths, the depth resolution of the system remains highly unequal across different distance planes. Under a uniform pitch configuration, the quadratic dependence on depth causes depth resolution to degrade rapidly at farther distances, resulting in non-uniform sensing performance throughout the volume [17,18,19,20,21,22,23]. To preserve approximately identical depth resolution across all target depths, the physical pitches of the array must be scaled non-uniformly. According to the approximate squared-multiple rule, the pitches must scale as squared multiples of the pitch at the closest depth (e.g., *p*, 4p, and 16p for objects located at relative depths of *z*, 2z, and 4z) during both the acquisition and computational reconstruction stages.

In this paper, we propose a computational reconstruction method for integral imaging that simultaneously eliminates shifting pixel quantization errors and preserves approximately uniform depth resolution across multiple target depths by using non-uniform camera array pitches. The proposed method calculates a single unified optimum pitch for multiple depths using a least common multiple (LCM) search over their respective optimum pitch bases. In parallel, the physical layout of the camera array is structured non-uniformly according to the approximate squared-multiple rule to maintain a constant depth resolution limit throughout the distance range. The performance of the proposed method is validated quantitatively and visually, demonstrating its potential for advanced sensing applications, such as autonomous driving, digital twins, and industrial robotics.

## 2. Computational Reconstruction and Quantization Errors

**Figure 1:**
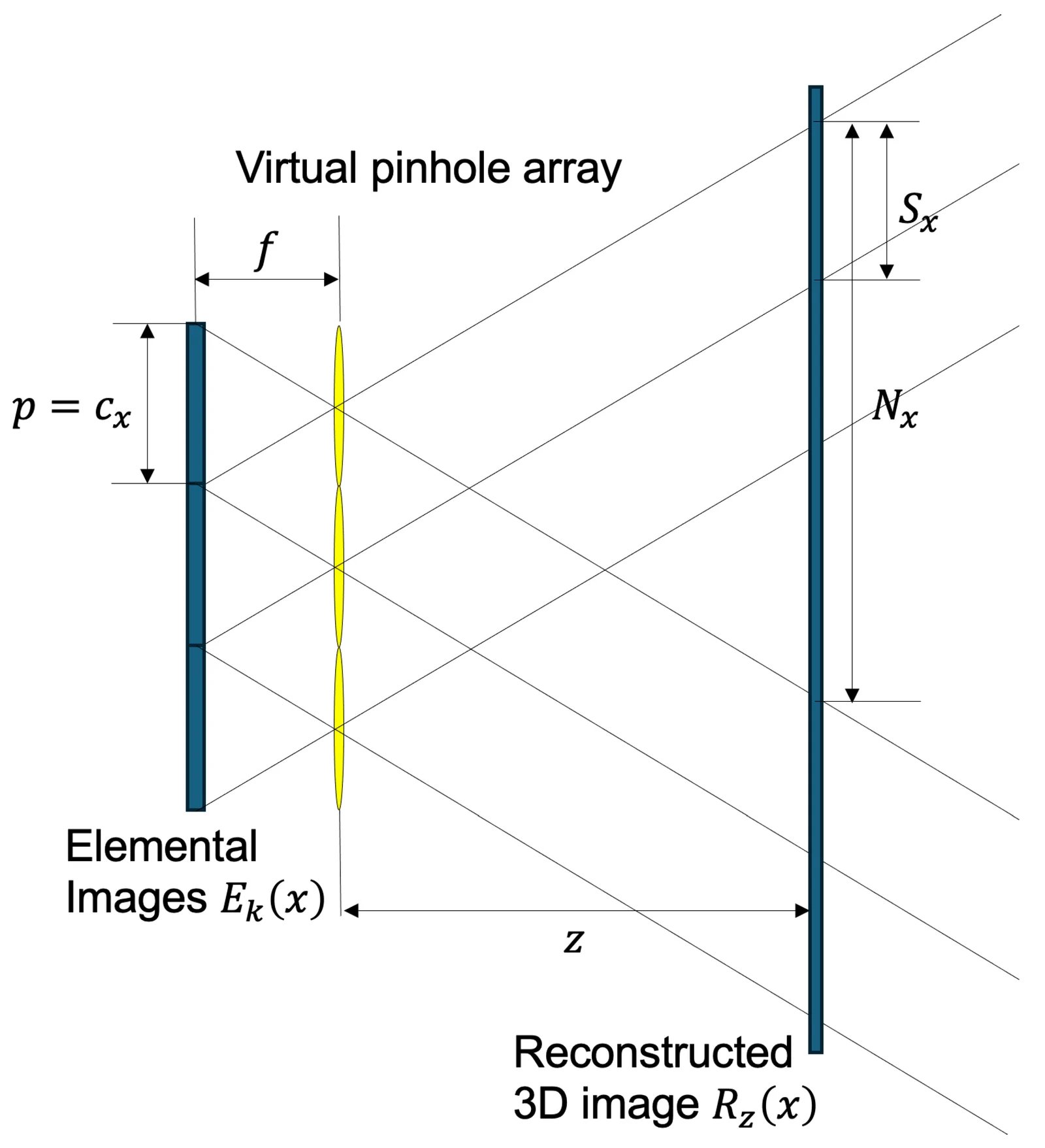
Schematic diagram of the VCR process in integral imaging, illustrating the back-projection of elemental images through a virtual pinhole array and their overlap on the reconstruction plane at depth *z*.

In integral imaging, a 3D image at specific reconstruction depths can be computationally reconstructed using digital processing techniques such as volumetric computational reconstruction (VCR) [11,12,13,14], as illustrated in Figure 1. To obtain these reconstructed 3D images, the acquired elemental images are computationally back-projected through a virtual pinhole array and superimposed on the target reconstruction plane at depth *z*. This digital process mathematically simulates physical optical reconstruction and enables the selective focusing of objects at different distance planes.

During digital back-projection, each elemental image is shifted by a calculated coordinate distance to align corresponding features on the reconstruction plane. The actual shifting pixel value, $S_{x}\left(k\right)$, which determines the lateral displacement of the coordinates throughout the array, is calculated as [11,12,13,14]:

(1) $$S_{x}=\frac{N_{x}fp}{c_{x}z}$$

where $N_{x}$ represents the number of pixels in an elemental image, *f* is the focal length of the lenses or cameras, *p* is the pitch between adjacent lenses or cameras, $c_{x}$ is the overall sensor size and *z* is the target reconstruction distance. Therefore, the actual shifting pixel value for the *k*th elemental image is $S_{x}\left(k\right)=S_{x}\times k$.

Because VCR operates in a discrete digital domain, these actual shifting sequences must be quantized to integer values to align with the discrete pixel grid of the sensor. Depending on how quantization is applied, VCR can be categorized into two primary methods: fixed VCR [11,12] and non-uniform VCR (NVCR) [14].

In fixed VCR, a single rounded shifting pixel value is used to calculate the shifts for all elemental images [11,12]:

(2) $${\hat{S}}_{x}\left(k\right)=\left\lceil S_{x}\right\rfloor \times k=\left\lceil \frac{N_{x}fp}{c_{x}z}\right\rfloor \times k,\;\mathrm{for}\;k=0,1,2,\ldots ,L_{x}-1$$

where ⌈·⌋ denotes nearest-integer rounding, *k* is the elemental image index, and $L_{x}$ is the total number of elemental images in the *x* direction. Because this uniform rounding operation ignores fractional accumulation, a substantial cumulative quantization error develops at larger index values.

To minimize this cumulative quantization error, NVCR calculates an individually rounded pixel shift, ${\tilde{S}}_{x}\left(k\right)$ for each elemental image [14]:

(3) $${\tilde{S}}_{x}\left(k\right)=\left\lceil S_{x}\times k\right\rfloor =\left\lceil \frac{N_{x}fp}{c_{x}z}\times k\right\rfloor ,\;\mathrm{for}\;k=0,1,2,\ldots ,L_{x}-1$$

By applying localized rounding, NVCR significantly reduces the average quantization error.

In both fixed VCR and NVCR, the final reconstructed 3D images at depth *z* are obtained by averaging the shifted and overlapped elemental images over the normalized overlapping area [11,12,13,14]:

(4) $${\hat{R}}_{z}\left(x\right)=\frac{1}{{\hat{O}}_{z}\left(x\right)}\sum_{k=0}^{L_{x}-1}E_{k}\left(x+{\hat{S}}_{x}\left(k\right)\right)$$

(5) $${\tilde{R}}_{z}\left(x\right)=\frac{1}{{\tilde{O}}_{z}\left(x\right)}\sum_{k=0}^{L_{x}-1}E_{k}\left(x+{\tilde{S}}_{x}\left(k\right)\right)$$

where $E_{k}$ denotes the *k*th elemental image, and ${\hat{O}}_{z}\left(x\right),{\tilde{O}}_{z}\left(x\right)$ are the overlap matrices for fixed VCR and NVCR, respectively.

The average quantization errors per elemental image for fixed VCR ($Q_{F}$) and NVCR ($Q_{N}$) are expressed as follows:

(6) $$Q_{F}=\frac{1}{L_{x}}\sum_{k=0}^{L_{x}-1}\left|S_{x}\left(k\right)-{\hat{S}}_{x}\left(k\right)\right|=\frac{1}{L_{x}}\sum_{k=0}^{L_{x}-1}\left|\frac{N_{x}fp}{c_{x}z}\times k-\left\lceil \frac{N_{x}fp}{c_{x}z}\right\rfloor \times k\right|$$

(7) $$Q_{N}=\frac{1}{L_{x}}\sum_{k=0}^{L_{x}-1}\left|S_{x}\left(k\right)-{\tilde{S}}_{x}\left(k\right)\right|=\frac{1}{L_{x}}\sum_{k=0}^{L_{x}-1}\left|\frac{N_{x}fp}{c_{x}z}\times k-\left\lceil \frac{N_{x}fp}{c_{x}z}\times k\right\rfloor \right|$$

To verify Equations (6) and (7), we apply the benchmark system parameters at z=1000 mm shown in Table 1. Using Equation (1), the actual shifting pixel values are $S_{x}\left(k\right)=S_{x}\times k=\frac{1500\times 50\times 11.76}{36\times 1000}k=\frac{147}{6}k=\left[0,24.5,49,73.5,98,122.5,147,171.5,196\right]$. Using Equation (2), shifting pixel values of the fixed VCR are ${\hat{S}}_{x}\left(k\right)=25k=\left[0,25,50,75,100,125,150,175,200\right]$. The average quantization error is $Q_{F}=\frac{18}{9}=2$ pixels by Equation (6). For NVCR, each shift is rounded directly from the real-valued product, yielding ${\tilde{S}}_{x}\left(k\right)=\left[0,25,49,74,98,123,147,172,196\right]$ by Equation (3). The average quantization error $Q_{N}$ is approximately 0.222 pixels by Equation (7). Thus, NVCR reduces the average quantization error by approximately 90% compared to fixed VCR.

## 3. Optimum Pitch Design for Zero Quantization Error

Although NVCR reduces shifting pixel quantization error, it may not reduce the error to exactly zero because individual shifts are still constrained to integer values. To achieve a zero quantization error ($Q_{F}=Q_{N}=0$), the actual shifting pixels must resolve to exact integers [15].

To establish the condition for perfect reconstruction, we require the base shifting pixel value between the first and second elemental images (i.e., k=1) to equal exactly one pixel [15]:

(8) $$\frac{N_{x}fp_{0}}{c_{x}z}=1\to p_{0}=\frac{c_{x}z}{N_{x}f}$$

where $p_{0}$ is the optimum pitch basis. Any pitch that is an integer multiple (*n*) of this basis also yields zero quantization error during reconstruction [15]:

(9) $$p_{opt}=np_{0},\;n\in Z^{+}$$

where $Z^{+}$ denotes the set of positive integers.

We verify the optimum pitch basis at target depth 1 (z=500 mm) using the benchmark system parameters shown in Table 1. The optimum pitch basis is calculated as $p_{0}=0.24$ mm by Equation (8). By choosing the system pitch $p=p_{0}=0.24$ mm, the actual shifting pixel sequence becomes $S_{x}\left(k\right)=k$. Since every actual shift $S_{x}\left(k\right)=k$ is already an integer, we have ${\hat{S}}_{x}\left(k\right)={\tilde{S}}_{x}\left(k\right)=k$ in both fixed VCR and NVCR. As a result, the shifting pixel quantization error for both VCR and NVCR is identically zero ($Q_{F}=Q_{N}=0$). This mathematical proof confirms that matching the physical spatial pitch of the array with the scene depth eliminates the shifting pixel quantization errors during computational reconstruction.

## 4. Optimum Pitch for Multiple Object Depths

Because the optimum pitch basis is directly proportional to depth *z*, a uniform array can satisfy the zero quantization error condition for specific depth planes which have an integer shifting pixel value. If a 3D scene contains multiple objects distributed at different depths $z_{1},z_{2},\ldots ,z_{n}$, a uniform pitch array may not optimize all planes simultaneously.

To resolve this limitation, we propose finding a single unified optimum pitch that satisfies the integer shifting pixel criterion at all target depths simultaneously. Let the individual optimum pitch bases for target depths $z_{1},z_{2},\ldots ,z_{n}$ be [15]:

(10) $$p_{i}=\frac{c_{x}z_{i}}{N_{x}f},\;\mathrm{for}\;i=1,2,\ldots ,n$$

The unified optimum pitch $p_{opt}$ is defined as the least common multiple (LCM) of these individual pitch bases:

(11) $$p_{opt}=\mathrm{LCM}\left(p_{1},p_{2},\ldots ,p_{n}\right)$$

To demonstrate the proposed method, we set three target depths at $z_{1}=500$, $z_{2}=1000$, and $z_{3}=2000$ mm under the benchmark system parameters shown in Table 1. First, we calculate the individual pitch bases $p_{1}=0.24$, $p_{2}=0.48$, and $p_{3}=0.96$ mm by Equation (10). Then, we find the unified optimum pitch $p_{opt}=LCM\left(0.24,0.48,0.96\right)=0.96$ mm by Equation (11). However, our proposed method determines the optimum pitch and non-uniform camera layout only after defining the recording depth range. To prevent the absence of LCM, we employ approximate pitches that ensure the existence of LCM.

Using $p_{opt}=0.96$ mm, we verify the actual shifting pixel values ($S_{xi}$) for each individual target depth $S_{x1}=4$, $S_{x2}=2$, and $S_{x3}=1$ pixels by Equation (1). Because all calculated shifting pixel values are exact integers, zero quantization error is achieved at all three target depths simultaneously during reconstruction.

To confirm that the unified optimum pitch preserves depth accuracy, we verify that these exact shifting pixels recover the original target depths by Equation (1) as:

(12) $$z_{1}=\frac{1500\times 50\times 0.96}{36\times 4}=500\;\mathrm{mm}$$

(13) $$z_{2}=\frac{1500\times 50\times 0.96}{36\times 2}=1000\;\mathrm{mm}$$

(14) $$z_{3}=\frac{1500\times 50\times 0.96}{36\times 1}=2000\;\mathrm{mm}$$

These exact analytical calculations prove that the LCM method achieves an exact recovery of multiple depths without shifting pixel quantization error.

## 5. Preserving Uniform Depth Resolution

Even when an optimum pitch satisfies the integer shift condition at multiple depths, the system’s depth resolution varies substantially across distance planes, resulting in unequal sensing performance.

**Figure 2:**
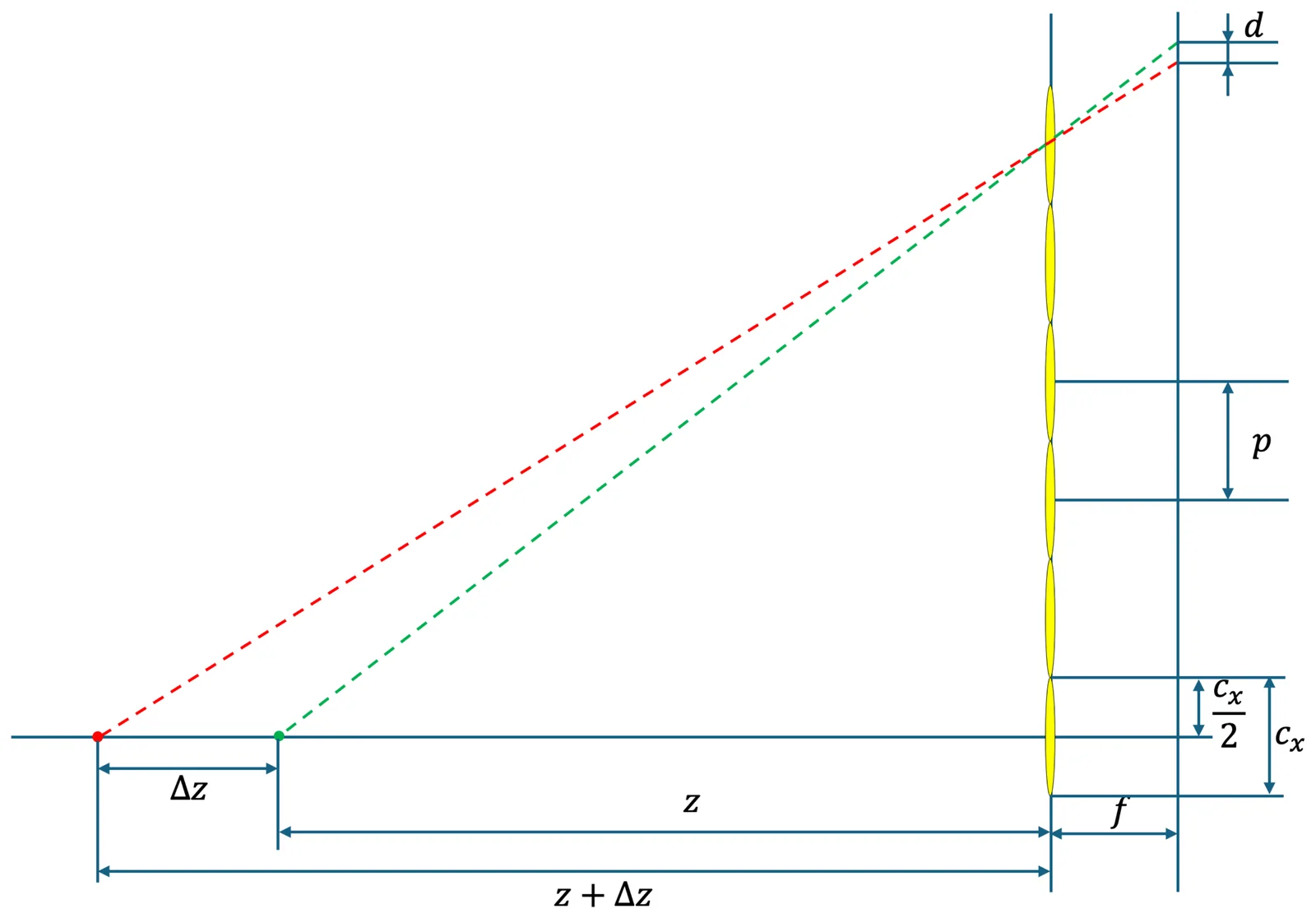
Depth resolution of integral imaging.

To analyze depth resolution, we examine the geometric relationship among target depth *z*, physical pitch *p*, lens focal length *f*, total number of elemental images $L_{x}$, and sensor pixel size $c=c_{x}/N_{x}$. The disparity *d* between the projection of an object point at depth *z* and a shifted point at z+Δz on adjacent sensors must be at least the pixel size of the sensor *c* for the points to be resolvable, as shown in Figure 2. The physical depth resolution limit Δz is therefore [17]:

(15) $$\Delta z\geq \frac{c_{x}z^{2}}{N_{x}\left(L_{x}-1\right)pf-c_{x}z}$$

Applying the calculated uniform pitch $p_{opt}=0.96$ mm, we evaluate the depth resolution limit at each target distance as $\Delta z_{1}\approx 16.129$, $\Delta z_{2}\approx 66.6667$, and $\Delta z_{3}\approx 285.714$ mm using Equation (15). This numerical verification highlights a critical drawback. Under a uniform pitch layout, the depth resolution limit degrades quadratically ($z^{2}$), increasing from 16.129 mm at 500 mm to 285.714 mm at 2000 mm, causing severe defocus and sensing blur at farther distances.

To preserve identical depth resolution at all distances, we impose the constraint $\Delta z_{i}=\Delta z_{1}$. Let $p_{i}^{\prime }$ be the updated physical pitch required at depth $z_{i}$. Solving for $p_{i}^{\prime }$ gives the following:

(16) $$p_{i}^{\prime }={\left(\frac{z_{i}}{z_{1}}\right)}^{2}p+\frac{c_{x}\left(z_{1}z_{i}-z_{i}^{2}\right)}{z_{1}N_{x}\left(L_{x}-1\right)f}$$

We apply Equation (16) to calculate the pitch required at $z_{2}=1000$ mm and $z_{3}=2000$ mm relative to the closest plane $z_{1}=500$ mm with p=0.96 mm as $p_{2}^{\prime }\approx 4p$ and $p_{3}^{\prime }\approx 16p$, respectively. This derivation reveals a specific principle, which is referred to as the approximate squared-multiple rule in this paper. As implementing a non-uniform camera layout with exact non-uniform pitches may pose significant challenges, this paper employs the approximate squared-multiple rule. To preserve uniform depth resolution across multiple target depths, the physical pitches of the camera array must scale as the approximate squared-multiple of the depth relative to the closest plane.

**Figure 3:**
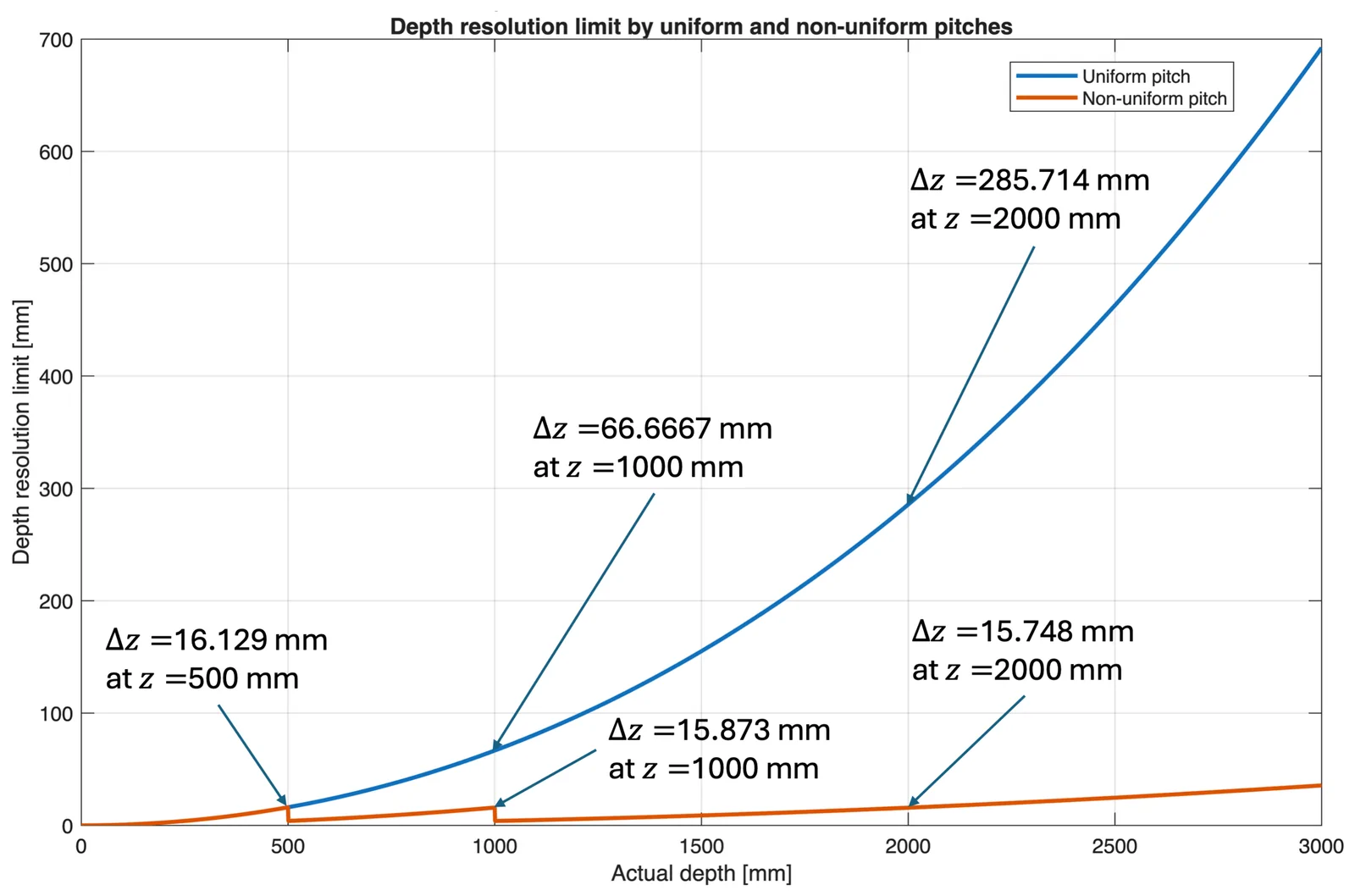
Comparison of depth resolution limit (Δz) between the conventional uniform pitch array (p=0.96 mm) and the proposed non-uniform pitch layouts (*p*, 4p, and 16p) based on the squared multiple rule. The proposed non-uniform layout maintains an approximately constant depth resolution of 16 mm across the distance range and avoids the quadratic degradation at farther distances, where N=1500, f=50 mm, p=0.96 mm, $c_{x}=36$ mm, and $L_{x}=9$.

We verify this non-uniform layout using pitches of p=0.96 mm, $p_{2}^{\prime }\approx 4p=3.84$ mm, and $p_{3}^{\prime }\approx 16p=15.36$ mm. The depth resolution limits are $\Delta z_{1}\geq 16.129$ mm, $\Delta z_{2}\geq 15.873$ mm, and $\Delta z_{3}\geq 15.748$ mm. Thus, the approximate squared-multiple rule maintains a virtually constant depth resolution (Δz≈16 mm) throughout the distance range, overcoming the quadratic resolution degradation, as shown in Figure 3.

## 6. Proposed Non-Uniform Pitch Method

**Figure 4:**
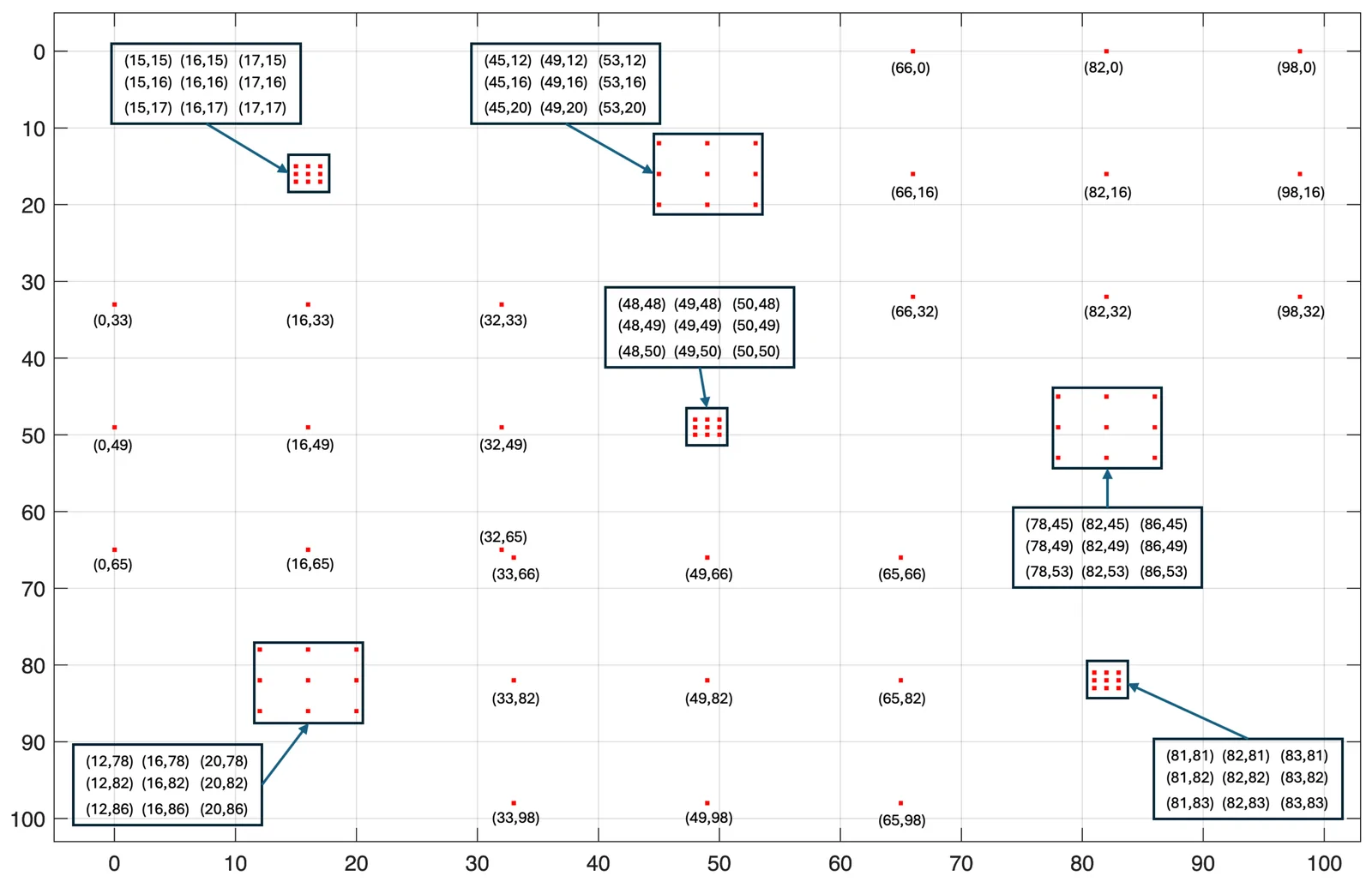
Proposed non-uniform pitch camera array layout using a Latin-square configuration of subarrays with pitches *p*, 4p, and 16p.

To physically implement the approximate squared-multiple rule, we propose a non-uniform camera array layout using a Latin-square spatial distribution. Instead of uniform distribution of cameras, we arrange the array as a 3×3 macro-array of subarrays, as illustrated in Figure 4. Each macro-element consists of a 3×3 camera subarray clustered at one of the calculated optimum pitches: p=0.96 mm, 4p=3.84 mm, or 16p=15.36 mm. To ensure a balanced spatial aperture, we arrange the pitches of these subarrays in a Latin-square configuration:

(17) $$P=\left[\begin{array}{ccc} p & 4p & 16p\\ 16p & p & 4p\\ 4p & 16p & p \end{array}\right]=\left[\begin{array}{ccc} 0.96 & 3.84 & 15.36\\ 15.36 & 0.96 & 3.84\\ 3.84 & 15.36 & 0.96 \end{array}\right]\mathrm{mm}$$

**Figure 5:**
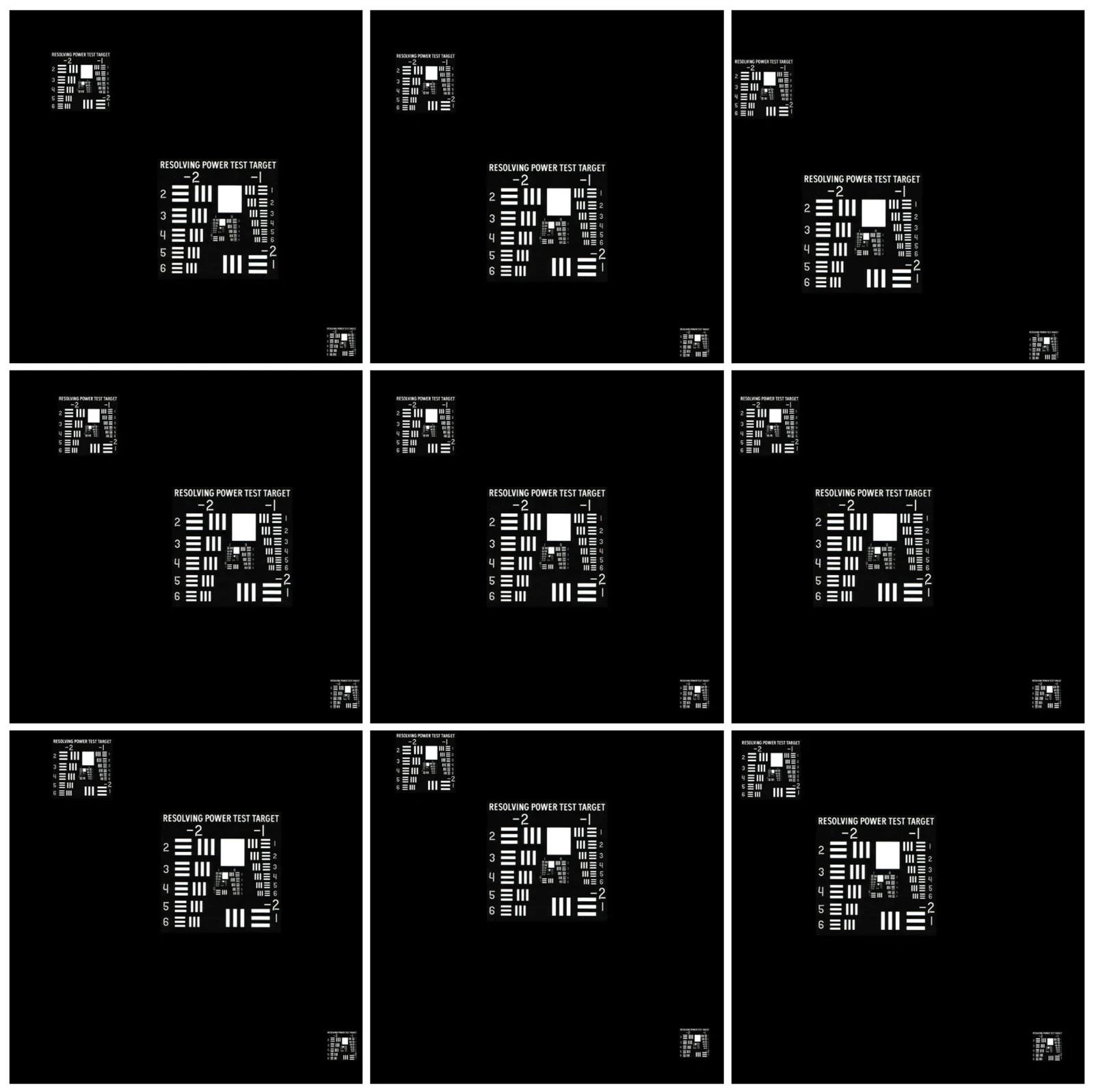
Elemental images captured by a camera array with the proposed non-uniform pitch method.

During pickup, the camera clusters simultaneously capture elemental images, as shown in Figure 5. Because the subarrays have distinct physical pitches, the captured elemental images are grouped. During computational reconstruction, each cluster group is mapped computationally using its matching pitch, ensuring that each object at its corresponding depth is reconstructed with zero quantization error and uniform depth resolution.

**Figure 6:**
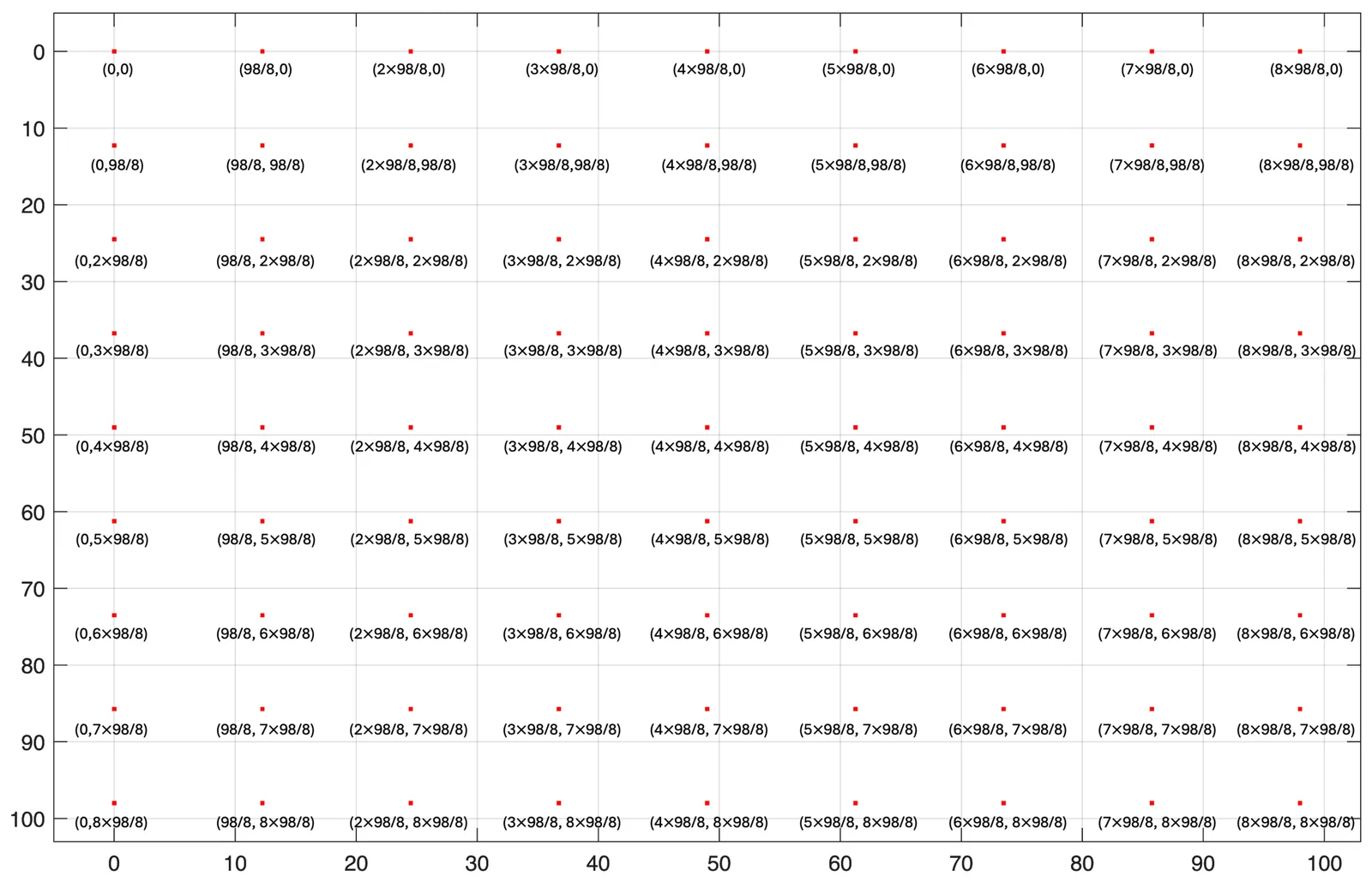
Uniform camera array layout using a pitch of 11.76 mm.

**Figure 7:**
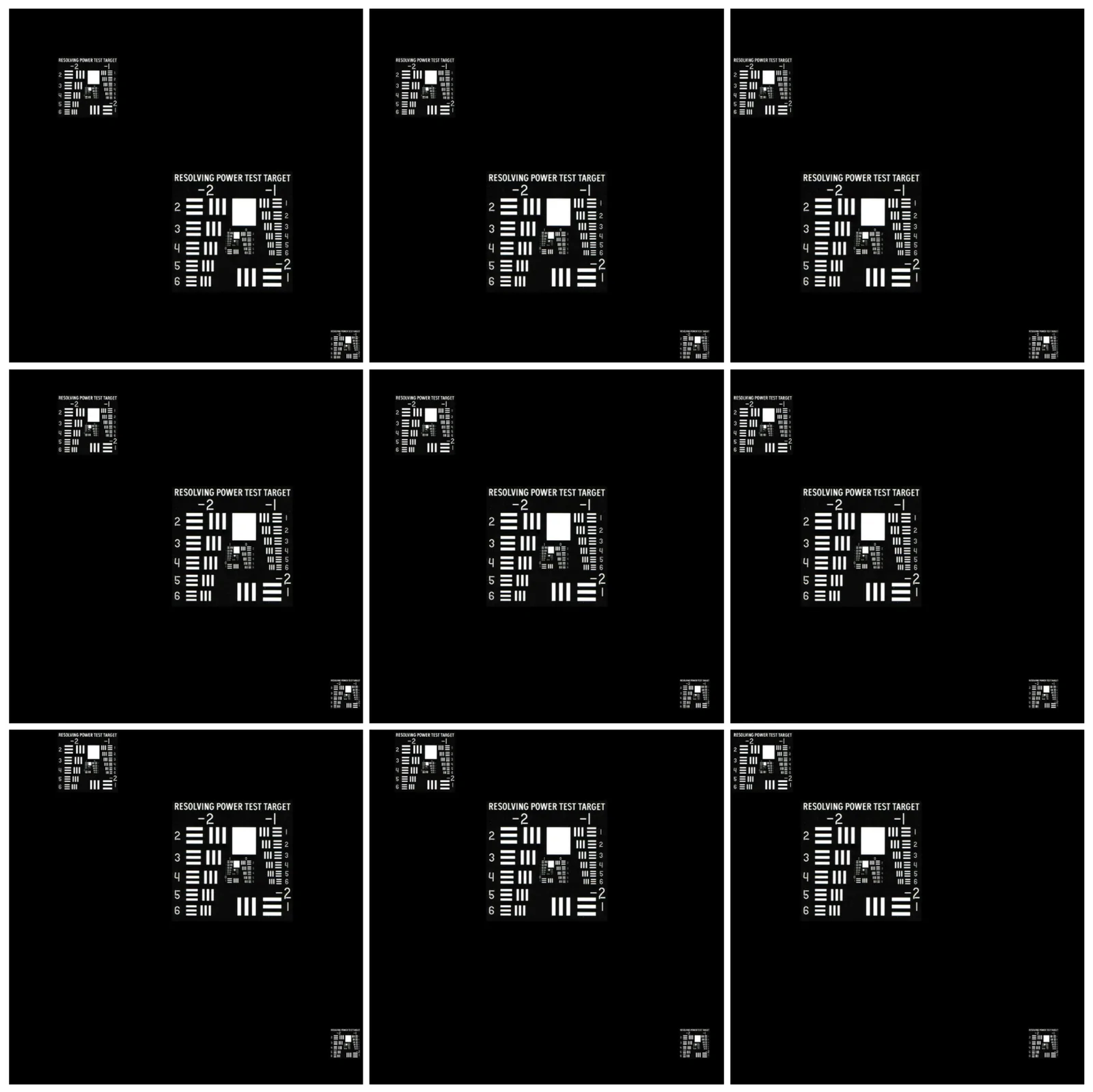
Elemental images captured by a uniformly distributed camera array with a pitch of 11.76 mm.

For comparison with the proposed method, we set the uniformly distributed camera array to a pitch of $\frac{98}{8}\times p=12.25p=12.25\times 0.96=11.76$ mm, as illustrated in Figure 6. Although the total pitch of this layout is the same as that of the proposed method, it cannot preserve depth resolution across all reconstruction depths. Figure 7 shows elemental images using the uniformly distributed camera array as mentioned above.

## 7. Simulation Results and Performance Evaluation

**Figure 8:**
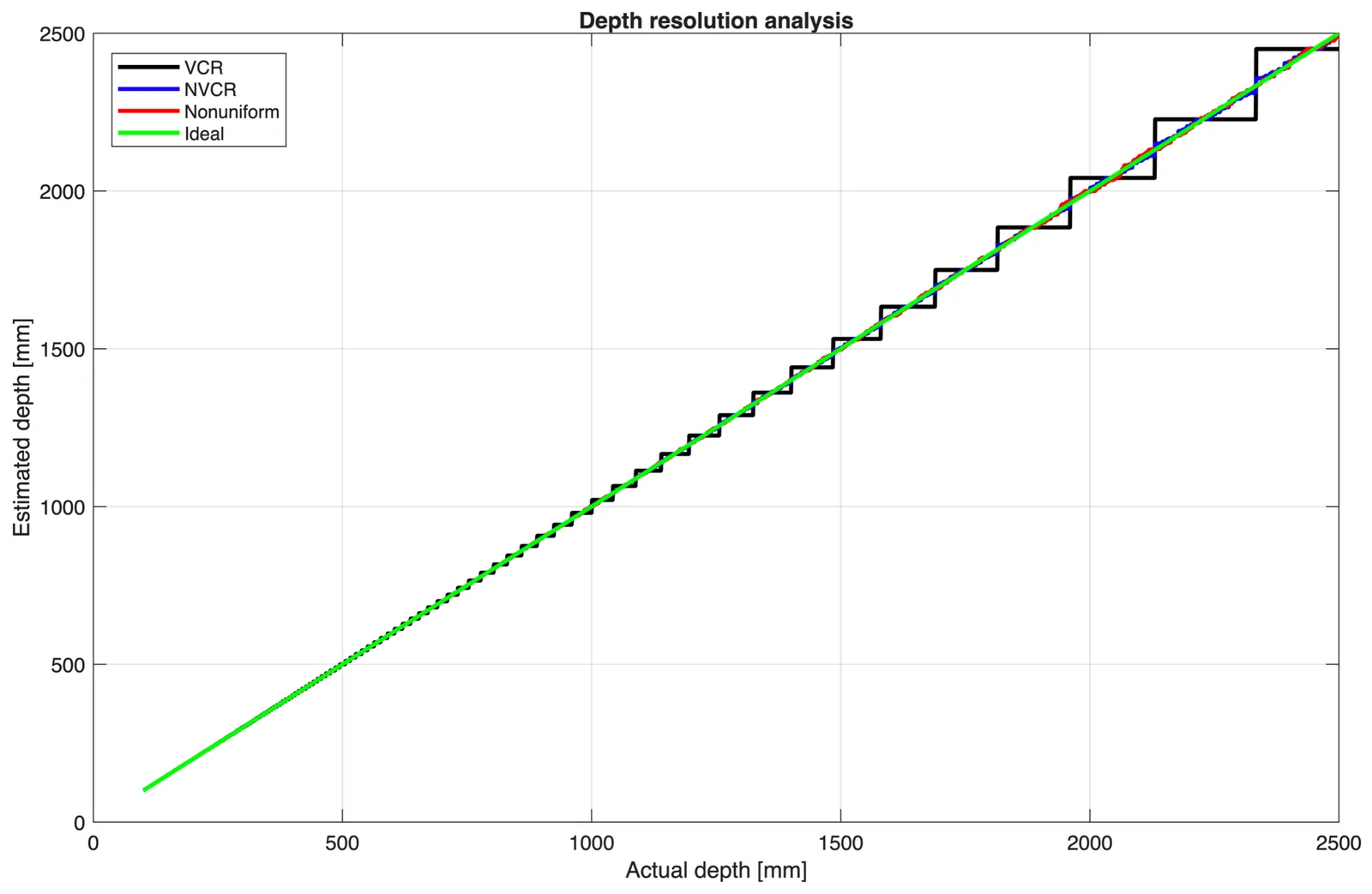
Depth estimation by fixed VCR, NVCR, and the proposed non-uniform pitch method across the full range (100–2500 mm) by using benchmark system parameters as shown in Table 1.

**Figure 9:**
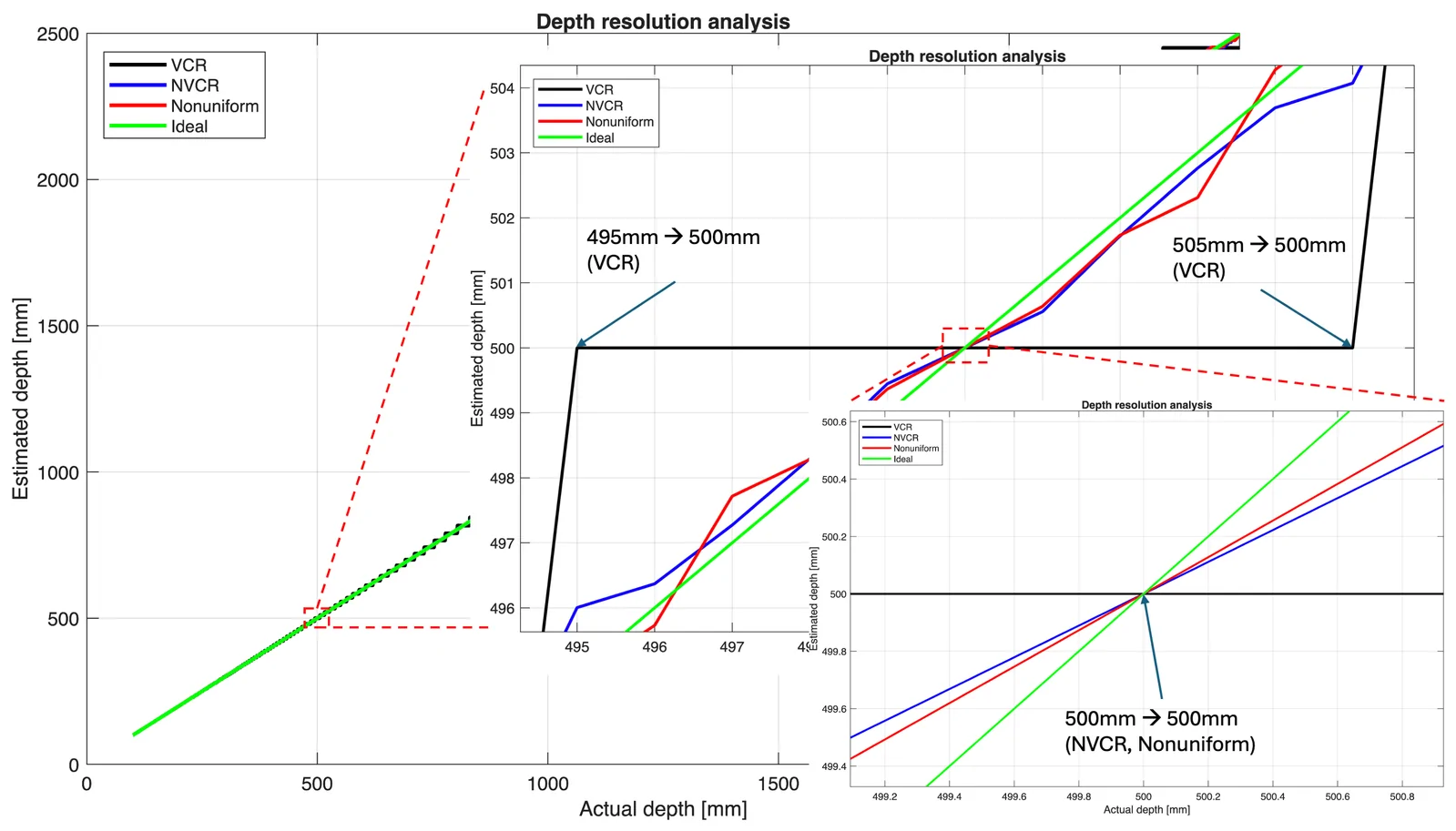
Depth estimation by fixed VCR, NVCR, and the proposed non-uniform pitch method across the full range (100–2500 mm) at target depth of 500 mm by using benchmark system parameters as shown in Table 1.

**Figure 10:**
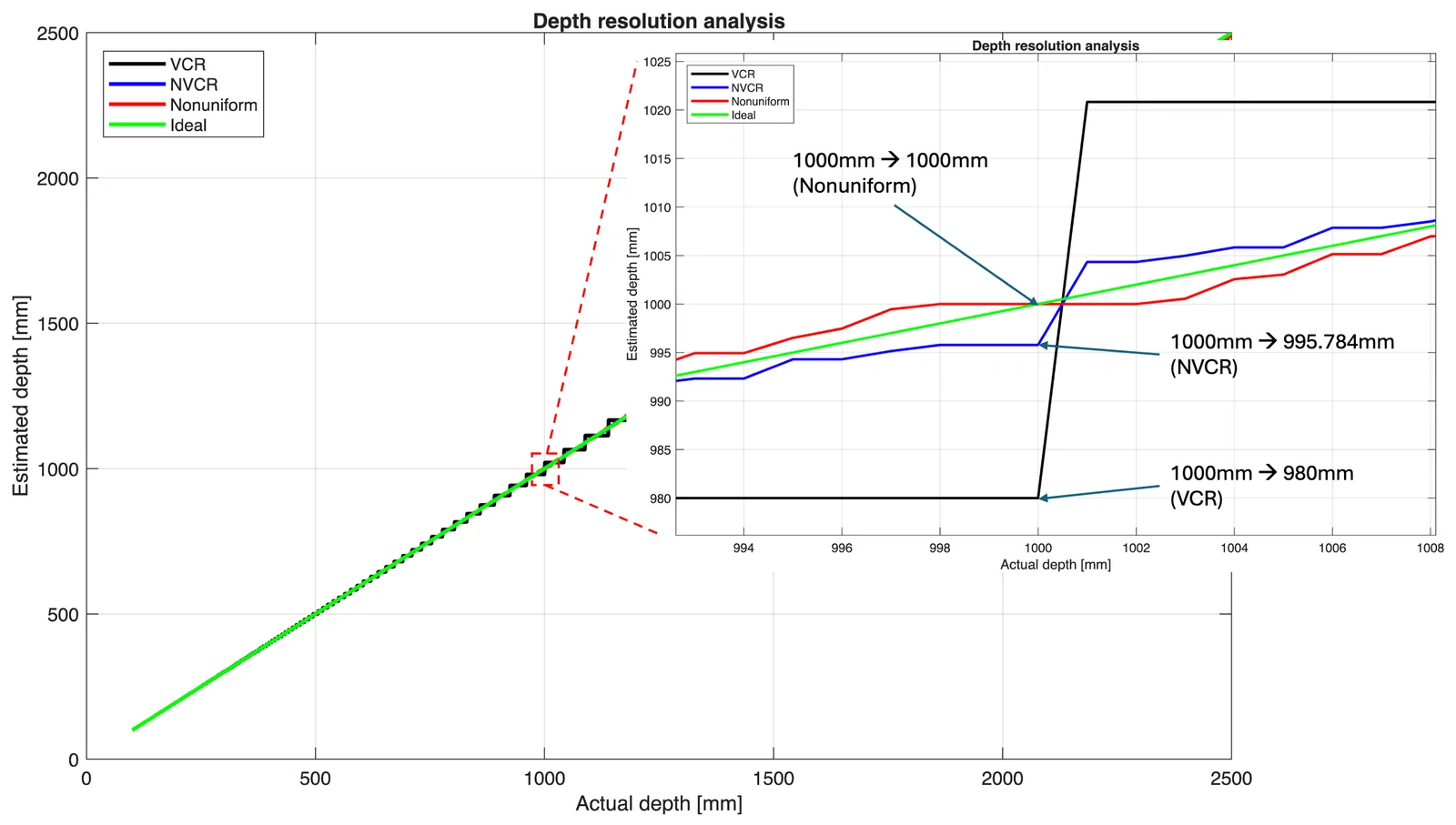
Depth estimation by fixed VCR, NVCR, and the proposed non-uniform pitch method across the full range (100–2500 mm) at target depth of 1000 mm by using benchmark system parameters as shown in Table 1.

**Figure 11:**
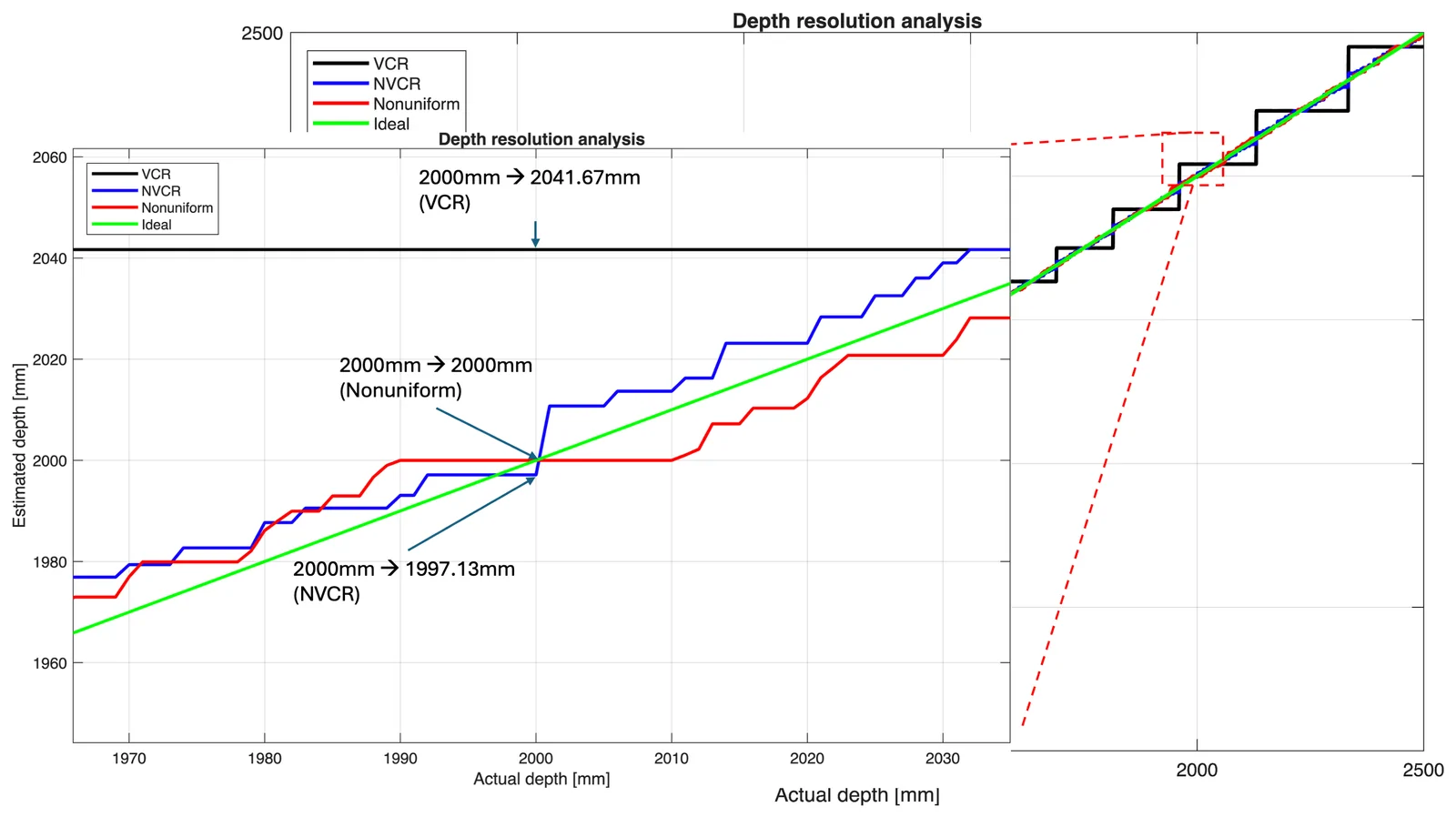
Depth estimationby fixed VCR, NVCR, and the proposed non-uniform pitch method across the full range (100–2500 mm) at target depth of 2000 mm by using benchmark system parameters as shown in Table 1.

To evaluate the proposed method, we conducted a computational simulation using the benchmark parameters shown in Table 1. USAF-1951 resolving-power test targets were placed at target depths of $z_{1}=500$, $z_{2}=1000$, and $z_{3}=2000$ mm. Figure 8, Figure 9, Figure 10 and Figure 11 show the depth estimation results across the full depth range, at 500, 1000, and 2000 mm, respectively. As shown in Figure 8, NVCR and the proposed method are almost identical to the ideal estimation (i.e., a linear function of y=x). However, at each target depth, as shown in Figure 9, Figure 10 and Figure 11, the proposed method shows a better estimation performance than the other methods.

We compared the estimated depths recovered using fixed VCR, NVCR, and the proposed non-uniform pitch method numerically. The recovered depths are shown in Table 2. As shown in Figure 9, both NVCR and our proposed method accurately estimate the target depth of 500 mm, whereas the fixed VCR yields an estimate of 495 mm. Similarly, in Figure 10, our proposed method achieves an exact estimate of 1000 mm, in contrast to the fixed VCR and NVCR, which estimate 980 mm and 995.784 mm, respectively. In Figure 11, while the fixed VCR and NVCR fail to provide accurate measurements, our proposed method precisely estimates the 2000 mm target depth. To further evaluate the robustness of our method at non-optimized depths, we performed tests at 800, 1300, and 2900 mm. As shown in Table 2, our proposed method demonstrates better accuracy compared to the alternative methods.

To analyze the depth estimation performance, we used the mean squared error (MSE) and the correlation coefficient for linearity, as shown in Table 3. Our proposed method yielded the lowest MSE and the highest linearity. Therefore, our method is shown to provide better depth estimation accuracy over a continuous depth range than the alternative approaches.

We also analyzed reconstruction focal sharpness quantitatively by plotting the peak sidelobe ratio (PSR) of the *k*th-law non-linear correlation results [24] for the reconstructed 3D images over a depth range from 400 mm to 2400 mm, where the non-linearity factor is set to k=0.5. The *k*th-law non-linear correlation filter is expressed as [24]

(18) $$r\left(x\right)=F^{-1}\left[{\left\{\left|I_{ref}\left(\mu \right)\right|\left|I_{s}\left(\mu \right)\right|\right\}}^{k}\exp\left\{i\left(\phi_{ref}\left(\mu \right)-\phi_{s}\left(\mu \right)\right)\right\}\right]$$

where $I_{ref}\left(\mu \right),I_{s}\left(\mu \right)$ are the Fourier transform of the reference and the observed image, *k* is nonlinearity factor, and $\phi_{ref}\left(\mu \right),\phi_{s}\left(\mu \right)$ are the phase of the reference and the observed image, respectively. We also define PSR value as the following:

(19) $$PSR=\frac{\max[\left(r\left(x\right)\right]-\bar{r}\left(x\right)}{\sqrt{\mathrm{var}[r(x)]}}$$

where $\bar{r}\left(x\right)$ denotes the mean of the correlation output r(x).

**Figure 12:**
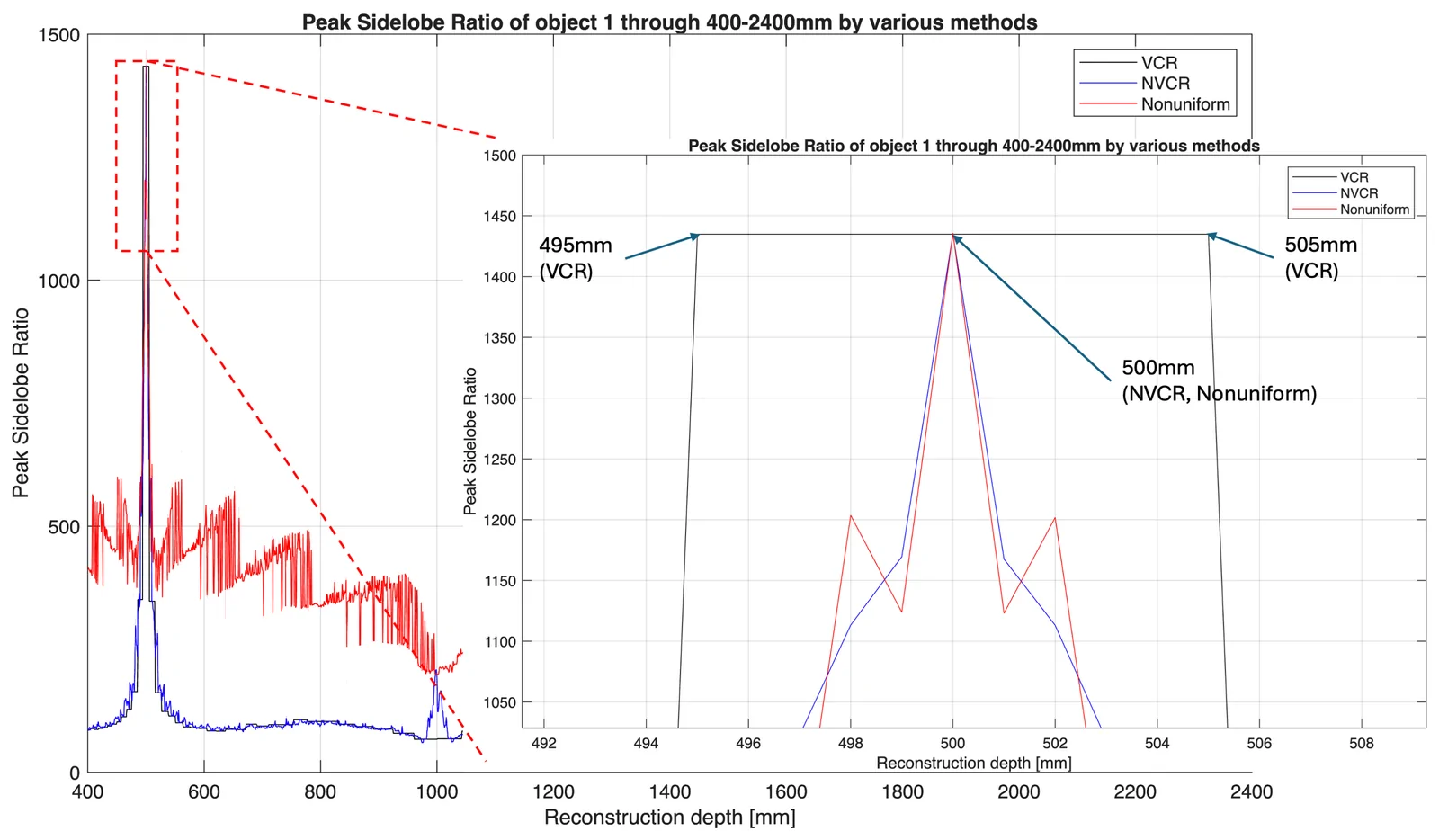
Peak sidelobe ratio for object 1 at z=500 mm by fixed VCR, NVCR, and non-uniform pitch method.

**Figure 13:**
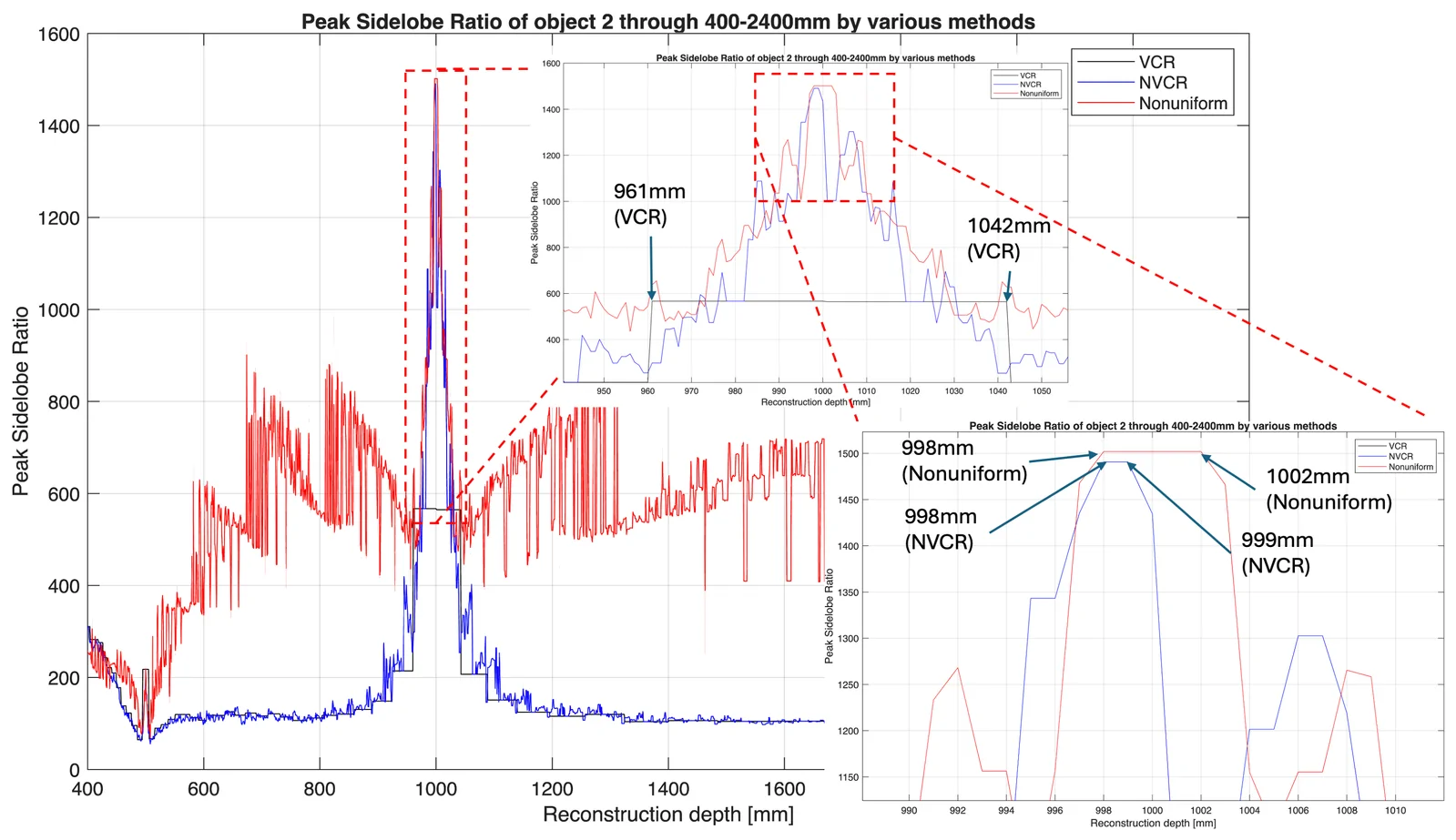
Peak sidelobe ratio for object 2 at z=1000 mm by fixed VCR, NVCR, and non-uniform pitch method.

**Figure 14:**
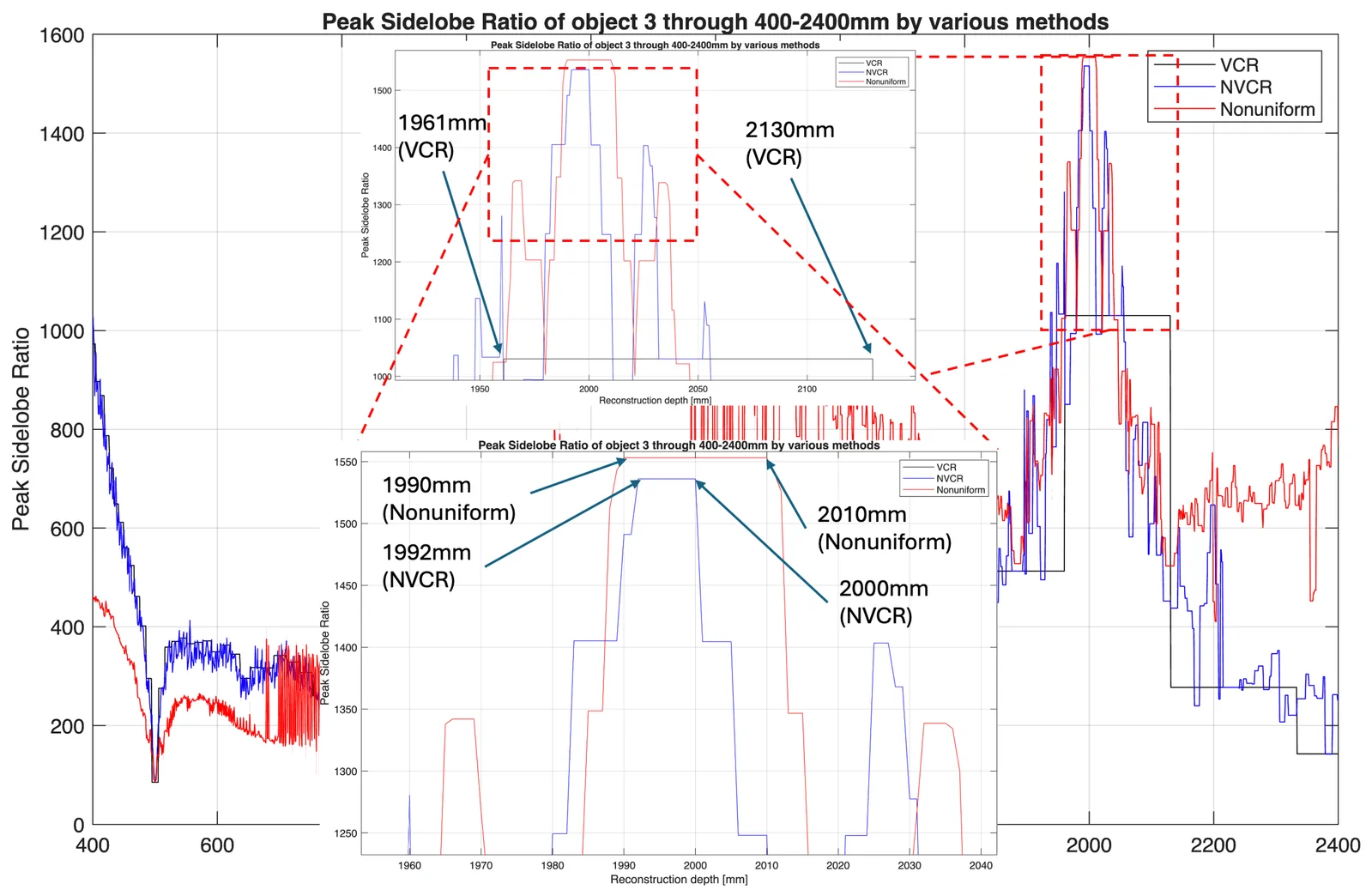
Peak sidelobe ratio for object 3 at z=2000 mm by fixed VCR, NVCR, and non-uniform pitch method.

The proposed non-uniform cluster method produces narrow, high-intensity PSR peaks precisely at target depths of 500, 1000, and 2000 mm, as shown in Figure 12, Figure 13 and Figure 14. In contrast, fixed VCR and NVCR show broader, shifted, or split peaks due to quantization-induced defocus.

At the first target depth $z_{1}=500$ mm, NVCR and the proposed method estimate the correct depth, while VCR produces a wide and inaccurate estimated depth range of 495–505 mm as shown in Figure 12. However, all methods satisfy the depth resolution Δz at 500 mm because it is approximately ±16 mm. At the second target depth $z_{2}=1000$ mm, fixed VCR produces an even wider estimated depth range of 961–1000 mm, with a median depth of 981 mm, as shown in Figure 13. It is noticed that both the depth resolution and the depth estimation accuracy of fixed VCR deteriorate significantly as the target depth increases. NVCR also estimates an inaccurate depth because it does not produce its highest PSR value at 1000 mm. In contrast, the proposed method estimates the correct depth while preserving approximately the same depth resolution as at the first target depth (approximately ±16 mm). Finally, as shown in Figure 14, at the third target depth $z_{3}=2000$ mm, the estimated target depths of fixed VCR, NVCR, and the proposed method are 2045.5 mm, 1995 mm, and 2000 mm, respectively. Therefore, these results demonstrate that our proposed method can accurately estimate the target depth while maintaining uniform depth resolution across the entire depth range.

**Figure 15:**
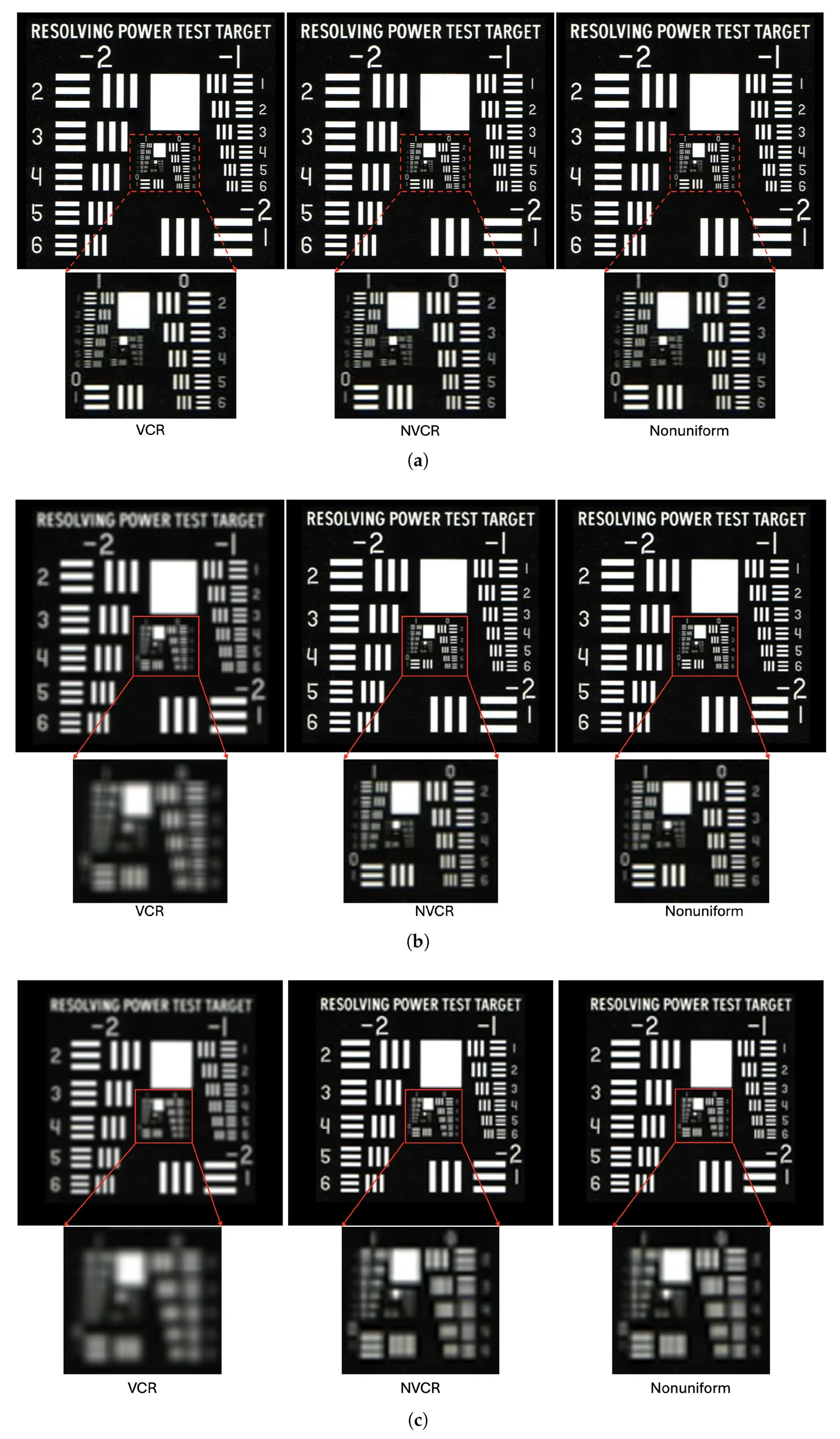
Comparison of reconstructed 3D images by fixed VCR, NVCR, and non-uniform pitch method for (**a**) object 1 (z=500 mm), (**b**) object 2 (z=1000 mm), and (**c**) object 3 (z=2000 mm).

Figure 15 shows the reconstructed 3D images at 500, 1000, and 2000 mm using fixed VCR, NVCR, and the proposed non-uniform pitch method. In Figure 15a, the visual quality of the reconstructed 3D images is identical across all methods because object 1 is placed at z=500 mm and the shifting pixel quantization error for all methods is exactly zero. In contrast, as shown in Figure 15b,c, the visual quality of the reconstructed 3D images at z=1000 mm and z=2000 mm by fixed VCR is worse than that of the other methods because its shifting pixel quantization error is larger.

Quantitative reconstruction performance was evaluated using the mean squared error (MSE) at each target depth. The results are summarized in Table 4. Across all target depths, the proposed non-uniform pitch method remarkably achieves error metrics of exactly zero, such as average quantization error and MSE, whereas fixed VCR and NVCR show non-zero average quantization error and MSE values. At 1000 mm and 2000 mm target depths, fixed VCR has non-zero average quantization error and MSE. NVCR has non-zero average quantization error and MSE at 1000 mm, and has non-zero average quantization error at 2000 mm. The reason why the proposed method has zero error metrics across all target depths is that the proposed method uses the optimum pitches according to the squared multiple rule so that the shifting pixel value is integers at any target depth. Therefore, the visual quality of the reconstructed 3D images can be improved and the uniform depth resolution can be preserved across all target depths.

The proposed method achieves an MSE of exactly zero at all target depths. At farther distances, fixed VCR and NVCR show noticeable reconstruction errors because of shifting pixel quantization mismatch.

## 8. Conclusions

In this paper, we proposed a computational reconstruction method for integral imaging that completely eliminates shifting pixel quantization errors while preserving uniform depth resolution across multiple target depths. We obtained a unified optimum pitch ($p_{opt}$) for multiple depths using an LCM search over their individual optimum pitch bases. However, our proposed method does not account for cases where no LCM exists for the optimum pitches, or where the LCM is too large for practical implementation. We intend to investigate these issues in future work. To address the quadratic degradation of depth resolution at farther distances, we formulated the approximate squared-multiple rule and implemented it physically using a Latin-square distribution of camera subarrays. Mathematical analysis and computational simulations showed that the proposed non-uniform pitch method achieves exact depth recovery with zero MSE and produces sharp, high-intensity PSR responses. The proposed non-uniform pitch method provides a robust and practical design for advanced spatial sensing technologies, such as autonomous driving, digital twins, and industrial robotics. However, it is necessary to consider environments with dynamic depth ranges, as the camera layout must be adjusted accordingly. We plan to investigate this aspect in future research.

## Figures and Tables

**Table 1:** Benchmark system parameters.

Parameter	Symbol	Value
Sensor pixel resolution	$N_{x}$	1500 pixels
Focal length of lens or camera	*f*	50 mm
Sensor size	$c_{x}$	36 mm
Number of lenses or cameras	$L_{x}$	9
Target depth 1	$z_{1}$	500 mm
Target depth 2	$z_{2}$	1000 mm
Target depth 3	$z_{3}$	2000 mm
Reconstruction range	z	100–2500 mm
Reconstruction step	$z_{s}$	1 mm
Pitch of fixed VCR and NVCR	$p_{f}=p_{v}$	11.76 mm
Pitch of the proposed non-uniform pitch	$p_{n}$	0.96, 3.84, 15.36 mm

**Table 2:** Comparison of reconstructed depth recovery (mm).

Target Depth	VCR Recovery	NVCR Recovery	Proposed Method Recovery
500 (optimized depth)	495	500	500
1000 (optimized depth)	980	995.784	1000
2000 (optimized depth)	2041.17	1997.13	2000
800 (non-optimized depth)	790.323	798.779	799.008
1300 (non-optimized depth)	1289.47	1297.05	1300.26
2900 (non-optimized depth)	3062.5	2919.42	2900.31

**Table 3:** Mean squared error (MSE) and Linearity for various reconstruction methods.

Reconstruction Method	MSE	Linearity
Fixed VCR	1036.601708	0.9989293235
NVCR	24.096641	0.9999749534
Proposed non-uniform pitch	11.554669	0.9999879867

**Table 4:** Comparison of reconstruction performance and error metrics.

Target Depth	Reconstruction Method	Physical Pitch (*p*)	Shifting Pixel ($S_{x}$)	Average Quantization Error	MSE
500 mm	Fixed VCR	11.76 mm	49 (Integer)	0.0000	0.0000
500 mm	NVCR	11.76 mm	49 (Integer)	0.0000	0.0000
500 mm	Proposed non-uniform pitch	0.96, 3.84, 15.36 mm	4, 16, 64 (Integer)	0.0000	0.0000
1000 mm	Fixed VCR	11.76 mm	24.50	2.0000	0.0740
1000 mm	NVCR	11.76 mm	24.50	0.2222	0.0000
1000 mm	Proposed non-uniform pitch	0.96, 3.84, 15.36 mm	2, 8, 32 (Integer)	0.0000	0.0000
2000 mm	Fixed VCR	11.76 mm	12.25	1.0000	0.0641
2000 mm	NVCR	11.76 mm	12.25	0.2222	0.0000
2000 mm	Proposed non-uniform pitch	0.96, 3.84, 15.36 mm	1, 4, 16 (Integer)	0.0000	0.0000

## Data Availability

Dataset available on request from the authors.

## Abbreviations

The following abbreviations are used in this manuscript:

2D	Two-dimensional
3D	Three-dimensional
LCM	Least common multiple
MSE	Mean squared error
NVCR	Non-uniform volumetric computational reconstruction
PSR	Peak sidelobe ratio
VCR	Volumetric computational reconstruction
